# The *Rhodococcus opacus* TadD protein mediates triacylglycerol metabolism by regulating intracellular NAD(P)H pools

**DOI:** 10.1186/1475-2859-12-104

**Published:** 2013-11-09

**Authors:** Daniel P MacEachran, Anthony J Sinskey

**Affiliations:** 1 Department of Biology, Massachusetts Institute of Technology, 31 Ames Street Bldg. 68-370, Cambridge, MA 02142, USA; 2 Department of Health Sciences & Technology, Massachusetts Institute of Technology, 31 Ames Street Bldg. 68-370, Cambridge, MA 02142, USA; 3 Department of Engineering Systems Division, Massachusetts Institute of Technology, 31 Ames Street Bldg. 68-370, Cambridge, MA 02142, USA; 4 Biochemical Products Group, Logos Technologies, Inc, 19955 Highland Vista Dr. Suite 130, Ashburn, VA 20147, USA

**Keywords:** *Rhodococcus opacus* PD630, GapN, triacylglycerol

## Abstract

**Background:**

The Gram-positive actinomycete *Rhodococcus opacus* is widely studied for its innate ability to store large amounts of carbon in the form of triacylglycerol (TAG). Several groups have demonstrated that *R. opacus* PD630 is capable of storing anywhere from 50 to 76% of its cell dry weight as TAG. While numerous studies have focused on phenomenological aspects of this process, few have sought to identify the underlying molecular and biochemical mechanisms responsible for the biosynthesis and storage of this molecule.

**Results:**

Herein we further our previous efforts to illuminate the black box that is lipid metabolism in actinomycetes using a genetic approach. Utilizing a simple, colorimetric genetic screen, we have identified a gene, referred to herein as *tadD* (*t*riacylglycerol *a*ccumulation *d*eficient), which is critical for TAG biosynthesis in *R. opacus* PD630. Furthermore, we demonstrate that the purified protein product of this gene is capable of oxidizing glyceraldehyde-3-phosphate, while simultaneously reducing NAD(P)^+^ to NAD(P)H. Supporting this biochemical data, we observed that the ratio of NAD(P)H to NAD(P)^+^ is elevated in wildtype cultures grown under lipid production conditions as compared to cultures grown under vegetative growth conditions, while the mutant strain demonstrated no change irrespective of growth conditions. Finally, we demonstrate that over-expressing a putative phosphorylative glyceraldehyde-3-phosphate dehydrogenase leads to decreased TAG production during growth on TAG accumulation conditions.

**Conclusion:**

Taken together, the data support the identification of a key metabolic branch point separating vegetative growth and lipid accumulation lifestyles in *Rhodococcus*.

## Background

The vast majority of organisms are capable of storing excess carbon and often do so when other nutrients are limiting, i.e. nitrogen, phosphorous, etc. [1-3]. Amongst bacteria, this stored carbon exists primarily as polyhydroxyalkanoate, though there are examples of other polymeric storage compounds including glycogen and complex, long-chain hydrocarbons [4-9]. Several actinomycetes, including *Mycobacterium tuberculosis* and several species of both *Streptomyces* and *Rhodococcus* have been shown to store excess carbon in the form of triacylglycerol (TAG) [10-14]. Indeed, *R. opacus* PD630 has previously been shown to accumulate up to 76% of its cell dry weight (CDW) as TAG when grown under nitrogen limiting conditions [13,14]. While several studies have been performed to characterize TAG biosynthesis and storage in *R. opacus* PD630 the underlying molecular and biochemical mechanisms remain poorly understood [12,15,16]. Work by the Sinskey lab has previously detailed the use of a Sudan Black based genetic screen to identify genes which mediate the biosynthesis and storage of TAG in *R. opacus* PD630 [17]. This work identified a novel protein, termed TadA, which mediates lipid storage in *R. opacus*. Furthermore, work by Alvarez and colleagues has identified the enzymes Atf1 and Atf2 which mediate late stages of the TAG biosynthetic pathway [15,18].

**Figure 1:**
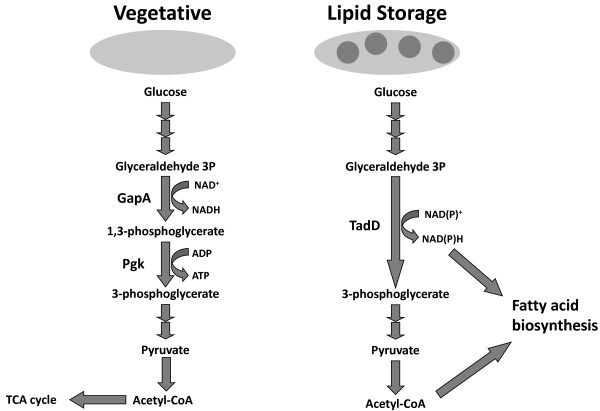
**Proposed model for the role of TadD in TAG metabolism in *R. opacus *PD630.** Under high nitrogen conditions (depicted on the left) glyceraldehyde 3-phosphate is metabolized to 3-phosphoglycerate by the canonical glyceraldehyde 3-phosphate dehydrogenase GapA and the phosphoglycerate kinase Pgk yielding NADH and ATP. We propose that under nitrogen limiting conditions G3P is metabolized to 3-phosphoglycerate by the non-phosphorylating, NAD(P)^+^-dependent glyceraldehyde 3-phosphate dehydrogenase TadD yielding NAD(P)H, a key redox molecule in fatty acid metabolism.

The non-phosphorylative glyceraldehyde 3-phosphate dehydrogenase (NP-G3P) family of enzymes, a sub-family of the larger aldehyde dehydrogenase family, was originally associated with green eukaryotes, plants and algae primarily, wherein it catalyzes the irreversible oxidation of glyceradehyde-3-phosphate (G3P) to 3-phosphoglycerate (3PG) while concomitantly reducing NAD(P)^+^ to NAD(P)H [19]. This reaction is mediated by the GapN protein in most organisms. This is in contrast to the canonical phosphorylative glyceraldehyde 3-phosphate dehydrogenase (GapA) which oxidizes G3P to 1,3-bisphosphoglycerate (1,3BPG) while reducing NAD^+^ to NADH. 1,3-BPG is subsequently dephosphorylated to 3-PG by the enzyme phosphoglycerate kinase yielding one molecule of adenosine triphosphate (ATP) (Figure 1) [20]. Thus instead of yielding one molecule of NADH and ATP, as would be the case in the phosphorylative branch of glycolysis, the non-phosphorylative branch yields a single molecule of NAD(P)H, an essential reducing equivalent in most biosynthetic pathways including fatty acid biosynthesis. While this family of enzymes was initially associated strictly with green eukaryotes, sequence and functional homologs have been identified in a number of eubacteria and archaea [21-23].

In this study we performed a genetic screen to identify genes that play a role in TAG biosynthesis and accumulation in *R. opacus* PD630. One of the mutants isolated in this screen has a transposon insertion in the 5′ end of a gene predicted to encode an aldehyde dehydrogenase. Utilizing a variety of techniques we demonstrate that the encoded product possesses NAD(P)H-dependent glyceraldehyde 3-phosphate dehydrogenase (ND-G3PD) activity. We propose that the predicted ND-G3PD enzyme is necessary for the generation of NAD(P)H utilized in the biosynthesis of fatty acids. Furthermore, our data suggests that activation (or derepression) of this protein may constitute an early switch from a vegetative lifestyle to a storage one. While several studies have identified genes that play a role in the later stages of TAG biosynthesis, we believe this study may be the first to identify one of the initial steps in this process.

## Results

### Genetic screen for *t*riacylglycerol *a*ccumulation *d*eficient (tad) mutants

**Figure 2:**
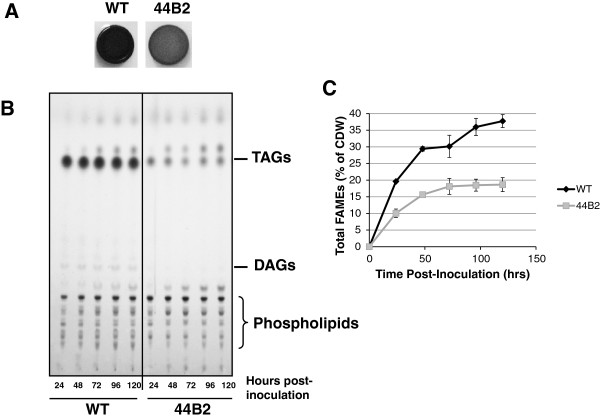
**The *R. opacus *PD630 44B2 transposon mutant accumulates less triacylglycerides (TAGs) than the wildtype. A**. Sudan Black staining of wildtype (WT) and 44B2 colonies grown on minimal medium supplemented with 4% (w/v) glucose and 0.15% (w/v) ammonium sulfate. The lipophylic dye Sudan Black was used to identify transposon mutants that accumulated less TAGs than the wildtype strain. Strains accumulating less TAGs absorb and retain less of the dye than the wildtype strain as can be seen in the 44B2 mutant colony. **B**. Thin-layer chromatography of the wildtype and 44B2 mutant grown for varying lengths of time. Lipid extracts from the two strains from progressive time points were resolved using a two solvent system to separate TAGs from other lipid species followed by charring. TAG standards were used to identify the Rf value for TAGs under the chromatographic conditions used. The 44B2 mutant accumulates less TAGs than the wildtype at all time points while the relevant abundance of other lipids remains largely unchanged. **C**. Biomass from kinetic assays was subjected to transesterification and the resulting FAMEs were separated via gas chromatography and detected using FID. The resulting spectrum was quantified against a standard curve of methyl esters. The wildtype strain produced more lipids than the 44B2 mutant at all time points reaching a maximum of 37.74% of total cell dry weight at 120 hours post inoculation as compared to the 44B2 mutant which peaked at 18.66% lipids.

The lipophylic dye Sudan Black was used to screen a library of 5000 Ez-Tn5 *R. opacus* mutants [17]. Mutants were grown under nitrogen limiting conditions on solidified minimal medium supplemented with 4% glucose and 0.15% ammonium sulfate followed by staining with Sudan Black. As previously described, TAG accumulation is evidenced by very dark blue staining of a colony as can be seen in the wildtype example from MacEachran *et al.*, 2010 and as shown in Figure 2A of this manuscript [17]. Mutants deficient in TAG accumulation stained a much lighter color as is shown for the 44B2 mutant (Figure 2A).

### Kinetics of TAG accumulation in the wildtype and 44B2 mutant strains

To better understand the kinetics of the TAG storage phenotype in strain 44B2 lipid storage was studied over time. The wildtype strain of *R. opacus* PD630, as well as the 44B2 mutant strain, was grown in minimal medium supplemented with 4% (w/v) glucose and 0.15% (w/v) ammonium sulfate. Cultures were sampled every 24 hours and several metrics were observed including glucose and nitrogen concentration, optical density at 600 nm, pH of the growth medium, the lipid content of the cells and the colony forming units. Interestingly, thin-layer chromatography (TLC) analysis demonstrated a marked decrease in neutral lipid accumulation in the 44B2 strain as compared to the wildtype strain (Figure 2B). There was no discernible difference in polar lipids between the two strains demonstrating that the defect seems to be specific to the biosynthesis of neutral, storage lipids. It is worth noting that the wildtype control shown in this experiment was previously published [17]. Densitometrical analysis of the TLC results suggests a 70-80% reduction in TAG accumulation in the mutant strain. Furthermore, there was no measurable difference in either glucose and nitrogen consumption nor colony forming units between the two strains suggesting no significant growth defect (data not shown).

To further confirm that the 44B2 mutant accumulated less lipids than the wildtype strain we utilized gas chromatography. Cell pellets from kinetic experiments identical to those described above were lyophilized and the total lipids extracted and converted to fatty acid methyl esters (FAMEs) for use in GC and subsequently detected using a Flame Ionization Detector (FID) (Figure 2C). Consistent with the TLC results, we found that after 120 hours of growth the mutant accumulated 18.66% (+/− 2.09%) lipids as a percentage of cell dry weight (mg lipids/mg CDW) while the wildtype accumulated 37.75% (+/− 1.95%) a difference of roughly 50%.

Utilizing a marker rescue-like approach we identified the transposon insertion site in the 44B2 mutant as lying 17 base pairs 3′ from the predicted start codon of a gene, termed herein *tadD*, predicted to encode a hypothetical protein with approximately 50% sequence identity to the aldehyde dehydrogenase family of proteins. The accession number for this protein is EHI47090 (encoded by OPAG_03892). Interestingly, this protein is predicted to contain a well-conserved NAD(P)^+^ binding domain.

### TadD has NAD(P)H-dependent glyceraldehyde 3-phosphate dehydrogenase activity

With the demonstration that loss of *tadD* expression resulted in a significant decrease in TAG accumulation we sought to better understand the biochemical underpinnings of the TadD protein. Bioinformatic analysis of the TadD protein suggested that it may possess NAD(P)H-dependent glyceraldehyde 3-phosphate dehydrogenase activity (ND-G3PDH). This family of proteins were initially identified in and thought to occur exclusively in the so-called green eukaryotes. More recently, ND-G3PDH homologs have been identified in several Gram-positive organisms [21,24,25].

**Figure 3:**
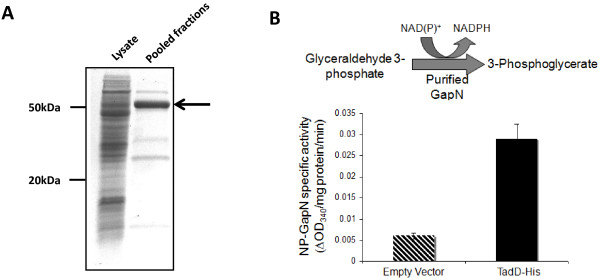
**Purified TadD demonstrates NAD(P)**^**+**^**-dependent glyceraldehyde 3-phosphate dehydrogenase (ND-G3PDH) activity. A**. SDS-PAGE analysis of purified TadD. A C-terminal hexa-histidine tagged variant of TadD was expressed recombinantly in *E. coli* from plasmid pDPM21 and purified using nickel affinity chromatography. The arrow marks the recombinant TadD protein. **B**. Purified TadD reduces NAD(P)^+^ in the presence of glyceraldehyde 3-phosphate (G3P). The purified histidine-tagged variant of TadD was incubated with G3P and NAD(P)^+^ and NAD(P)H formation was monitored as an increase in absorbance at 340 nm. Values are expressed as a change in absorbance at 340 nm per mg of protein per minute. We observed a marked increase in specific activity in fractions containing the TadD protein as compared to those from cells harboring the empty vector.

To assess whether the TadD protein possessed ND-G3PDH activity we constructed a C-terminal hexa-histidine tagged variant of the TadD protein, expressed it in *E. coli* and purified the protein utilizing nickel affinity chromatography (Figure 3A). Additionally, supernatant from *E. coli* containing the parental plasmid was used as an empty vector control. Fractions containing the histidine tagged TadD protein (and the corresponding fractions from the empty vector fractionation) were then used in ND-G3PDH activity assays. As shown in Figure 3B reactions containing the purified TadD protein generated more NAD(P)H than those containing extract from the empty vector strain as demonstrated by the increase in optical density at 340 nm suggesting that indeed TadD possesses ND-G3PDH activity.

### TadD ND-G3PDH activity is induced under lipid storage conditions

With the demonstration that the TadD protein possessed ND-G3PDH activity we sought to determine whether this activity was coordinated with lipid biosynthesis. As NAD(P)H is essential to numerous biosynthetic pathways, specifically fatty acid biosynthesis, we hypothesized that we would see an increase in TadD-dependent ND-G3PDH activity in cells that have begun to accumulate TAGs.

**Figure 4:**
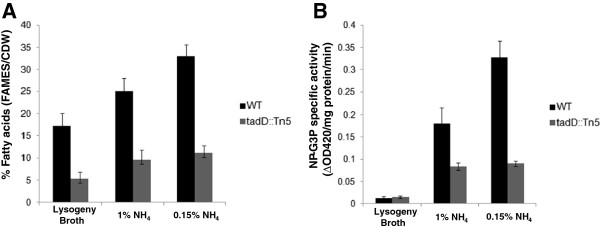
**TadD-dependent ND-G3PDH activity increases under lipid accumulation conditions in *R. opacus *PD630. A**. Lipid accumulation increases during nitrogen limiting conditions. Lipids were extracted from wildtype *R. opacus* PD630 and the *tadD* mutant grown in either LB or minimal medium supplemented with either 1% (w/v) or 0.15% (w/v) ammonium sulfate and assayed using GC-FAMEs analysis. As predicted we observed an increase in total fatty acids when wildtype *R. opacus* PD630 was grown under nitrogen limiting conditions as compared to nitrogen replete conditions. Additionally, the *tadD* mutant demonstrated decreased lipid accumulation as compared to the wildtype, as shown above. **B**. ND-G3PDH activity in crude lysates from cultures grown under vegetative and lipid storage conditions. Cultures from above were lysed and the crude lysates assayed for ND-G3PDH activity. As with the total fatty acids, we observed a significant increase in the ND-G3PDH activity in cells grown under nitrogen limiting conditions as compared to those grown under nitrogen replete conditions. Furthermore, we observed significantly lower ND-G3PH activity in the *tadD* mutant.

To assess whether the observed TadD ND-G3PDH activity was induced under lipid storage conditions we grew wildtype and the *tadD* transposon mutant in either rich LB medium (non TAG storage conditions) or MR medium supplemented with 4% (w/v) glucose and either 1% (w/v) (low TAG storage conditions) or 0.15% (w/v) (TAG storage conditions) ammonium sulfate for 24 hours. As we sought to determine whether TadD-dependent ND-G3PDH activity changed under TAG storage versus non-storage conditions we first needed to determine whether there was in fact any difference in lipid accumulation under these three different conditions. To this end we assayed for the total fatty acid content of cultures grown under these three conditions using GC-FAMEs followed by FID (Figure 4A). Wildtype *R. opacus* accumulated more fatty acids per mg of cell dry weight under the TAG storage conditions than either the high nitrogen or rich media conditions. Furthermore, as expected the *tadD* transposon mutant accumulated less fatty acids than the wildtype under all of the conditions tested.

These same cultures were then assayed for ND-G3PDH activity using the previously described assay. As we hypothesized, we saw a sharp increase in ND-G3PDH activity in cultures grown under lipid storage conditions (Figure 4B). Interestingly, the ND-G3PDH activity is superimposable over the GC-FAMES data with very low activity and fatty acid accumulation in the LB grown cultures, an intermediate phenotype for both metrics under the high nitrogen conditions and high ND-G3PDH activity and fat storage under the low nitrogen condition. It is worth noting that there does appear to be some residual ND-G3PDH activity in the *tadD* transposon mutant. This could be the result of either partial activity of the mutant *tadD* gene product or the result of another protein with redundant or promiscuous ND-G3PDH activity.

**Figure 5:**
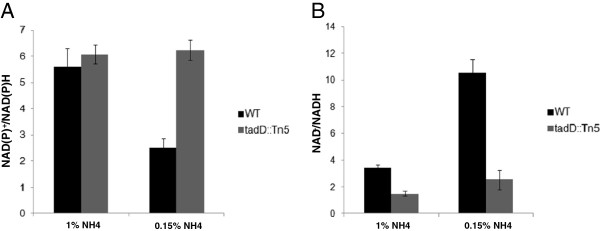
**NAD(P)+/NAD(P)H and NAD+/NADH ratios in the wildtype and *tadD *mutant. A**. NAD(P)^+^/NAD(P)H ratios decrease during early nitrogen limitation. Wildtype *R. opacus* PD630 and the *tadD* mutant were grown in minimal medium supplemented with either 1% (w/v) or 0.15% (w/v) ammonium sulfate, the resulting cell lysates were assayed for NAD(P)^+^ and NAD(P)H concentrations. Consistent with our data concerning ND-G3PDH activity we observed a decrease in the ratio of NAD(P)^+^/NAD(P)H under nitrogen limitation as compared to nitrogen replete conditions. Additionally, we see no change in the ratio of NAD(P)^+^/NAD(P)H in the *tadD* mutant. **B**. The ratio of NAD^+^/NADH increases under nitrogen limiting conditions. Cell lysates from above were also assayed for NAD^+^ and NADH concentrations. We observed a marked increase in the ratio or NAD^+^ to NADH under nitrogen limiting conditions as compared to nitrogen replete conditions, while no difference was observed in the *tadD* mutant. It is worth noting that the NAD^+^/NADH ratio in the *tadD* mutant is significantly lower than that observed for the wildtype.

While we have shown that TadD-dependent ND-G3PDH activity increases dramatically under conditions that promote lipid accumulation we wanted to determine if this translated into a change in the NAD(P)H and NADH pools within the cells. Accordingly, we grew wildtype *R. opacus* and the *tadD* transposon mutant in minimal medium supplemented with 4% (w/v) glucose and either 1% (w/v) or 0.15% (w/v) ammonium sulfate as described above. We then determined the concentration of NAD(P)^+^ and NAD(P)H (Figure 5A) or NAD^+^ and NADH (Figure 5B) which are expressed as the ratios of the oxidized form divided by the reduced form. Consistent with the changes in TadD-dependent ND-G3PDH activity described above we observed a dramatic decrease in the ratio of NAD(P)^+^ to NAD(P)H with a concomitant increase in the NAD^+^ to NADH ratio when comparing the wildtype strain grown under nitrogen replete conditions to wildtype grown under nitrogen limiting conditions. As expected we did not observe any difference in NAD(P)^+^/NAD(P)H or NAD^+^/NADH ratios for the *tadD* mutant.

### Over-expression of a potential GapA homolog results in a decrease in fatty acid accumulation

**Figure 6:**
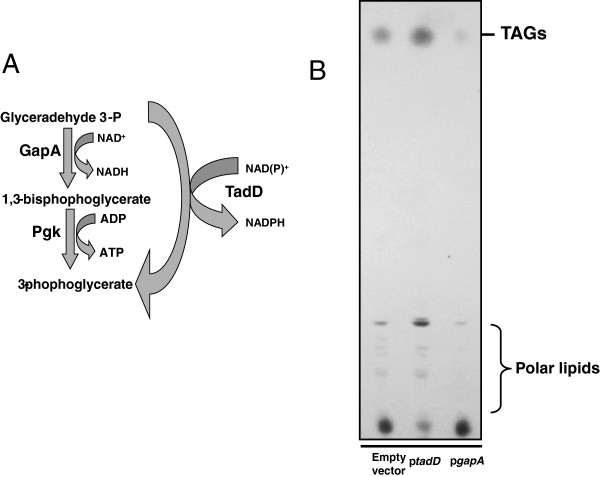
**Overexpression of a predicted *R. opacus *PD630 GapA homolog results in a decrease in TAG accumulation. A**. We hypothesize that GapA and GapN compete for G3P during glycolysis in *R. opacus* PD630. **B**. Extrachromosomal expression of GapA results in a decrease in TAG accumulation. Lipid extracts from wildtype *R. opacus* PD630 expressing either GFP, TadD or the *R. jostii* RHA1 GapA homolog encoded by the ro07177 gene grown in minimal medium supplemented with gentamycin were resolved using TLC. As described above, expression of the TadD protein resulted in an increase in TAGs while expression of the ro07177 protein resulted in a dramatic decrease in TAGs as compared to the wildtype strain containing the empty vector pDPM70.

Many bacteria metabolize glucose via either the Emden-Meyerhof or the Entner-Doudoroff pathway. One of the key energy generating steps of these pathways is the NAD^+^-dependent dehydrogenation of glyceraldehyde 3-phosphate by the canonical glyceraldehyde 3-phosphate dehydrogenase GapA, followed by a subsequent substrate level phosphorylation by phosphoglycerate kinase, yielding one molecule of NADH, ATP and 3-phosphoglycerate. NAD(P)^+^-dependent glyceraldehyde 3-phosphate dehydrogenases have been shown to bypass these two steps yielding a single molecule of NAD(P)H and 3-phosphoglycerate (Figure 6A). Based on our data we hypothesized that during vegetative growth, *R. opacus* PD630 metabolizes glucose and other hexoses via either of the two pathways described above utilizing the GapA-dependent pathway. However during lipid storage, we hypothesize that *R. opacus* switches to a GapN-dependent pathway thus yielding NAD(P)H, an essential cofactor in fatty acid biosynthesis. Accordingly, we hypothesized that over-expressing GapA should result in a decrease in available NAD(P)H and thus a decrease in lipid accumulation.

To this end we cloned a predicted *R. opacus* PD630 GapA homolog (homologous to the *R. jostii* ro07177 gene) into the *E. coli*/*Rhodococcus*/*Saccharomyces* expression vector pDPM70 and mobilized it into wildtype *R. opacus* PD630. Cultures of wildtype *R. opacus* containing the empty vector or expressing either the *gapA* homolog or *tadD* were grown in minimal medium supplemented with 4% (w/v) glucose, 0.15% (w/v) ammonium sulfate and gentamycin for 48 hours at 30°C. Fatty acid content was assayed via TLC (Figure 6B) and GC-FAMEs. We observed that, as predicted, over-expression of the GapA homolog resulted in a dramatic decrease in total fatty acids while over-expression of TadD resulted in an increase in TAG accumulation. These data are further supported by GC-FAMEs analysis with the empty vector strain containing 17.59% (+/−0.92%) total lipids (FAMEs/CDW), the *tadD* overexpressing strain containing 23.10% (+/− 1.59%) lipids and the *gapA* overexpressing strain containing 12.82% (+/− 0.84%) lipids. It is worth noting that, in general, the use of this plasmid system in *Rhodococcus* leads to overall decreases in lipid content within the host strain as can be observed in the difference in FAMEs between the wildtype strain harboring the empty vector (17.59% lipids per CDW) and the wildtype strain lacking any plasmid (29.4% lipids per CDW). At this time the cause of this reduction is not fully understood though it is believed that physiological stress of carrying the plasmid as well as the presence of antibiotics in the growth medium hamper lipid production and cellular growth. The inclusion of a vector control allows for normalization of these effects when studying the effects of gene expression on lipid biosynthesis.

## Discussion

Several species of actinomycetes have been shown to produce TAG, a process unique amongst bacteria [26-30]. Indeed, *R. opacus* PD630 has been shown to accumulate up to 76% of its cell dry weight as TAG when grown under nitrogen limiting conditions [14]. Similarly, *M. tuberculosis* has been shown to accumulate TAG in distinct, intracellular inclusion bodies when grown under nutrient limiting conditions, conditions similar to those believed to exist in the human lung during infection [31,32]. It is thought that these lipids are an important part of the pathogenic lifestyle in *M. tuberculosis*[32]. In addition to their potential role in pathogenesis, microbial lipids have garnered significant attention recently as potential feedstocks for the production of renewable green fuels including biodiesel, gasoline, diesel and jet fuel. The innate ability of *R. opacus* to produce large quantities of storage lipids, specifically TAG, makes this a model organism for the microbial conversion of lignocellulosic biomass to green, fungible fuels. By better understanding the underlying molecular and biochemical mechanisms mediating this process, more efficient strains and processes can be engineered.

Despite the importance of this pathway little work has focused on the molecular and biochemical processes underlying lipid production and storage in actinomycetes. Work by Alvarez *et al.* was the first to demonstrate that the acyltransferase protein Atf1 played a key role in lipid storage in *R. opacus* PD630 [15]. Indeed, disruption of this protein resulted in a 2-fold reduction in TAG content. Previous work by the Sinskey lab has demonstrated that the heparin-binding hemagglutinin TadA regulates lipid body maturation in *R. opacus* PD630. While the exact mechanism is not fully understood it is believed that the protein acts as an aggregation factor, leading to coalescence of nascent lipid bodies into larger mature storage inclusions [17]. Interestingly, recent work by Ding *et al.* has substantiated these findings by further demonstrating that the TadA protein is highly enriched in *R. opacus* lipid bodies where it is thought to play a role in maturation of lipid bodies [33]. It is worth noting that recent work by Pfeiffer and colleagues identified a protein in *Ralstonia eutropha* which is believed to work in a similar manner [34]. While the two proteins differ significantly in their sequence, there are striking similarities in their predicted structures and domains. Additionally, several studies have identified acyltransferase proteins that play a role in TAG biosynthesis in *M. tuberculosis*[11,29,35]. All of these studies have focused on the latter stages of TAG biosynthesis and storage. There is a relative dearth of knowledge regarding both the early steps of this process as well as the regulatory mechanisms that control TAG biosynthesis and storage in actinomycetes.

We have previously described a Sudan Black-based genetic screen that targeted lipid storage and biosynthesis related genes in *R. opacus* PD630 [17]. This work identified the aforementioned *tadA* gene, which as stated above, is believed to play a structural role in lipid body aggregation and maturation. This protein most likely acts in the later stages of lipid storage, a hypothesis that is supported by the relatively leaky TAG phenotype. In this work we continued to employ this genetic screen to investigate lipid biosynthesis in *R. opacus* PD630. We identified a mutant, 44B2, which demonstrated a ~50% reduction in lipid storage as compared to the wildtype strain. Mapping of the mutation responsible for this reduction identified a transposon insertion at the 5′ end of a gene predicted to encode an aldehyde dehydrogenase, which we named *tadD* for *t*riacylglycerol *a*ccumulation *d*eficient. Bioinformatic analysis of this gene and its predicted product demonstrated very weak sequence and structural homology to the family of non-phosphorylative glyceraldehyde 3-phosphate dehydrogenases (NP-G3PD), colloquially referred to as GapN proteins. NP-G3PD activity assays using purified TadD demonstrated higher, albeit relatively low, NAD(P)H generation than those performed using the empty vector strain (Figure 3B). Similar assays using whole cell lysates showed higher NP-G3PD activity in the wildtype than in the *tadD* transposon mutant (Figure 4B). Interestingly, the specific activity of the whole cell lysates was higher than that observed for the purified protein alone. While the mechanisms underlying this difference remain unknown it is tempting to speculate that there are additional factors present in the whole cell lysates that mediate activity of TadD. Alternatively, similar to other central carbon metabolism enzymes, it is possible that TadD is subject to allosteric activation *in vivo* and that the lack of this activation in *E. coli* results in a purified product that is not in its active state. Consistent with this hypothesis, previous work has demonstrated that the GapN protein from *Thermoproteus tenax* is subject to complex allosteric regulation [36]. It is worth noting that, as seen in Figure 4B, there was an increase in ND-G3PD activity observed in the *tadD* mutant grown on 1% (w/v) ammonium sulfate as compared to LB grown cultures. Furthermore, this activity was approximately 50% of that of the wildtype under the same conditions. The exact mechanism for this increased activity is not known. It could simply be that, as this is a transposon mutant, there is some level of expression of a truncated protein and that this basal level is the subject of allosteric regulation that effects its activity during lower nitrogen conditions. Alternatively, it is entirely possible that there are other proteins with some functional redundancy contained within the *R. opacus* 9.1 megabase genome, leading to this increased activity. Also of interest, this activity does not increase when the mutant strain is shifted from 1% (w/v) to 0.15% (w/v) ammonium sulfate while ND-G3PD activity increases dramatically in the wildtype. This further supports either model. At this time the mechanisms underlying this observation remain the focus of ongoing studies.

We hypothesized that the *R. opacus* TadD protein generates NAD(P)H essential for fatty acid metabolism and hence TAG production on a variety of carbon substrates. As most pentose and hexose sugars are metabolized via the second stage of glycolysis this pathway most likely plays a role in the conversion of a variety of metabolites to storage lipids. If this is the case, one would predict that TadD activity should increase in direct correlation to lipid biosynthesis. To test this hypothesis, we assayed TadD activity in cultures grown under vegetative and lipid storage conditions. Interestingly, as lipid storage increased (Figure 4A) we observed a concomitant increase in TadD activity (Figure 4B). As expected, both lipid storage and TadD activity were low in the *tadD* mutant under all conditions tested (Figures 4A and 4B). Following on these data we hypothesized that the increased TadD activity observed under lipid storage conditions should result in a decrease in the ratio of the oxidized vs. reduced form of NAD(P) (NAD(P)^+^/NAD(P)H) with a concomitant increase in the NAD^+^/NADH ratio during growth under lipid storage conditions. As seen in Figure 5, we observed a decrease in the ratio of NAD(P)^+^/NAD(P)H (Figure 5A) with an increase in the ratio of NAD^+^/NADH (Figure 5B) when comparing vegetative growth conditions (1% (w/v) ammonium sulfate) to lipid storage conditions (0.15% (w/v) ammonium sulfate). As expected, the *tadD* mutant did not display any change in either NAD(P)H or NADH ratios when comparing the two growth conditions. These data suggest that during the switch from a vegetative lifestyle to lipid storage the cell increases the biosynthesis of NAD(P)H, a key reducing equivalent for anabolic reactions, while simultaneously decreasing the reduction of NAD^+^ to NADH. Furthermore, the *tadD* mutant showed a relatively high ratio of NAD(P)^+^/NAD(P)H with a low ratio of NAD^+^/NADH suggesting that the cell was locked in this vegetative growth condition, an observation consistent with other data presented here.

Based on the *in vivo* activity data (Figure 4B) and the redox ratio data (Figures 5A and 5B) we hypothesized that there is a regulatory switch that is activated during the conversion from a vegetative lifestyle to a storage one. We hypothesized that during the vegetative growth phase, *R. opacus* metabolizes glucose via a standard glycolytic pathway, utilizing the phosphorylative GapA to oxidize G3P to 1,3-BPG which is subsequently subjected to a Pgk mediated substrate-level phosphorylation yielding one molecule of ATP and 3-PG (Figure 6A). However, during lipid storage the cell switches to the non-phosphorylative glycolytic pathway wherein TadD mediates the non-phosphorylative oxidation of G3P to 3PG (Figure 6B). If this is indeed the case, we would predict that over-expression of the canonical GapA protein should result in the maintenance of a vegetative lifestyle, marked by a relative lack of TAG, while over-expression of the TadD protein should result in an increase in TAG content as compared to the wildtype organism. Indeed, consistent with these results, over-expression of TadD in a wildtype background resulted in an increase in TAG while over-expression of GapA resulted in a decrease in total TAG. It is noteworthy that the TadD over-expressing strain demonstrated a slight decrease in total colony forming units as compared to the other two strains. Similar experiments were conducted using the 44B2 mutant strain with similar results (data not shown). However, it is worth noting that in this background, overexpression of the *tadD* gene did not fully restore the lipid content to wildtype levels. The underlying reason for this remains elusive but could be due to either underexpression of the gene from the plasmid system used, over-expression leading to insolubility of the protein or some form of post-translational allosteric regulation.

These data, taken together, suggest that the TadD protein plays an enzymatic role in TAG biosynthesis and storage. This contrasts the group’s previous data that demonstrated that the TadA protein most likely acts in a structural capacity, mediating lipid body aggregation and maturation. Overall, these two studies begin to illuminate the fact that the overall process of lipid biosynthesis and storage in *Rhodococcus* is most likely highly complicated with multi-tiered levels of regulation.

There is no doubt that the relative abundance of reduced NAD(P)H effects the biosynthesis of macromolecules in most organisms. Herein we present evidence that the regulation of the reduction of NAD(P)^+^ to NAD(P)H by the NADP^+^-dependent glyceraldehyde 3-phosphate dehydrogenase TadD is a key metabolic switch in the conversion from a vegetative growth phase to a lipid storage one in *R. opacus* PD630. We believe this is the first demonstration of the role in redox pools in regulating the switch from one mode of life to another.

## Materials and methods

### Bacterial strains, chemicals and media

All strains and plasmids used in this study are listed in Table 1. Bacteria were propagated in lysogeny broth [37,38] (Difco, Lawrence, Kansas) or minimal media unless otherwise noted. Minimal media was prepared as previously described [39] and supplemented with 4% (w/v) glucose and either 0.15% (w/v) or 1.0% (w/v) ammonium sulfate. Growth media was supplemented with kanamycin (100 μg/ml), gentamycin (10 μg/ml) or ampicillin (150 μg/ml). All restriction enzymes were purchased from New England Biolabs (Ipswich, MA). Chemicals were purchased from Sigma-Aldrich (St. Louis, MO).

**Table 1:** Strains, plasmids and primers used

**Strain**	**Relevant genotype**	**Source**
*Rhodococcus*		
*R. opacus* PD630	Wildtype strain	[39]
44B2	*tadD*::Tn5	This study
DPM245	WT + pDPM70	[17]
DPM278	WT + pDPM91	This study
DPM279	WT + pDPM92	This study
*E. coli*		
*E. coli* EC100D	*F*^*-*^*mcrA Δ(mrr-hsdRMS-mcrBC) φ80dlacZΔM15 ΔlacX74 araD139 Δ(ara, leu) pir-116(DHFR)*	Epicentre
DPM54	*E. coli* EC100D + pMQ70	This study
DPM55	*E. coli* EC100D + pDPM21	This study
*S. cerevisiae* INVSc1	*MATa his3D1 leu2 trp1-289 ura3-52*	Invitrogen
**Plasmids**		
pMQ70	oriMB1, oriT, *bla*, *araC*, P*araBAD*, CEN6, URA3	[44]
pDPM21	pMQ70 + *tadD*	This study
pDPM70	oriNG2, CEN6, *aacC1*, URA3, P*smyc*-*tetRO*	[17]
pDPM91	pDPM70 + ro07177	This study
pDPM92	pDPM70 + *tadD*	This study
**Primers**	**Sequence**	
pDPM21for	5′-GCTTGCATGCCTGCAGGTCGACTCTAGAG GATCCCCGGGTACCTTAATGATGATGATGAT GATGTCGTTTGACCCGCAGCACTCTGTAG-3′	
pDPM21rev	5′-CCGTTTTTTTGGGCTAGCGAATTCAGGAG GCTCTCTCTATGAGTATCGCCGCAGATTCTCT GTCC-3′	
pDPM91for	5′CGATCCGCTCGAGGCATGCAGAAAGGAGG CCATATGGGACTGCATGACTGTCCGGGTAGG CGTAAACGGTTTCGGCCG-3′	
pDPM91rev	5′-GCTATGACCATGATTACGCCAAGCTTGGT A CCGAGCTCGGATCAGAGAGACTTGGCGAC GAGACCGATGAGGTCG-3′	
pDPM92for	5′- CGATCCGCTCGAGGCATGCAGAAAGGAG GCCATATGGGACTGCATGAGTATCGCCGCAG ATTCTCTGTCC-3′	
pDPM92rev	5′- CAGCTATGACCATGATTACGCCAAGCTTGGTA CCGAGCTCGGATCATCGTTTGACCCGCAGCACTC TG-3′	

### Transposon mutagenesis and screening

The EZ-Tn5 transposome system from Epicentre Biotechnologies (Madison, WI) was used to introduce random mutations throughout the *R. opacus* PD630 genome [17]. Tn5 mutants were subsequently screened as previously described [17]. Briefly, Tn5 mutants were grown on minimal medium supplemented with 4% glucose and 0.15% ammonium sulfate for 120 hours. Following growth colonies were incubated with 0.1% Sudan Black in 95% EtOH for 15 minutes followed by washing in 95% EtOH. Lighter staining colonies were selected for further study. A marker rescue-like approach was used to map the chromosomal transposon insertion site [17].

### Lipid extraction and Thin-layer chromatography

Extraction of lipids from cultures was performed as previously described [17,40]. Briefly, cultures were centrifuged and the resulting pellets lyophilized. Lipids were extracted by adding a 1:1 chloroform:methanol solution to the dried pellets followed by incubation at room temperature for 60 minutes with agitation. Extracts were then filtered through a 0.2 μm PVDF filter to remove particulate matter. Thin-layer chromatography experiments were performed using a two-step resolution method as previously described [41,42]. 25 μg of lipid extract was spotted onto glass backed silica gel 60 TLC plates (EMD Chemicals Inc., Gibbstown, NJ) and dried under a constant stream of nitrogen. Samples were resolved using an initial polar buffer containing 60:35:5 chloroform:methanol:water, followed by a second buffer containing 70:30:1 hexane:diethyl ether:acetic acid. Plates were allowed to dry prior to charring by first exposing the plates to 3% cupric acetate in an 8% aqueous phosphoric acid solution followed by baking in a 200°C oven.

### Methanolysis and gas chromatography of lipids

Extraction of lipids and subsequent methanolysis to create fatty acid methyl esters (FAMEs) was performed as previously described [17,39,43]. Briefly, lyophilized cell pellets were resuspended in 1 ml of chloroform to which 1 ml of a 85:15 methanol:sulphuric acid solution was added. Samples were subsequently heated at 100°C for 2.5 hours followed by rapid cooling on ice. The organic phase was washed once with water and filtered through a 0.2 μm PVDF filter to remove any particulate matter. Gas chromatography of FAMEs (GC-FAMEs) was performed using an Agilent Technologies 6850 series II network GC system (Agilent Technologies, Santa Clara, CA) as previously described [39] and FAMEs were detected using a flame ionization detector (FID).

### Construction of plasmids pDPM21, pDPM91 and pDPM92

All plasmids were constructed using *Saccharomyces cerevisiae*-based homologous recombination cloning as previously described [17,44]. The TadD purification vector pDPM21 was constructed using primers pDPM21for and pDPM21rev. Full length *tadD* was amplified from the *R. opacus* PD630 chromosome and recombined into linearized pMQ70 [44].

The full length *R. jostii* ro07177 gene, a putative *gapA* homolog was amplified from the *R. jostii* RHA1 chromosome and recombined into the *Rhodococcus* / *E. coli* / *Saccharomyces* expression vector pDPM70 using primers pDPM91for and pDPM91rev to construct pDPM91 [17].

To construct the TadD expression vector pDPM92, the full length *tadD* gene was amplified from the *R. opacus* PD630 chromosome and recombined into the broad host range expression vector pDPM70 using primers pDPM92for and pDPM92rev.

Plasmid DNA was mobilized into *R. opacus* PD630 using electroporation as previously described [17].

### Purification of TadD

A C-terminal hexahistidine tagged variant of the TadD protein was purified using nickel affinity chromatography. Overnight cultures of *E. coli* EC100D containing either pMQ70 (empty vector) or pDPM21 were diluted 1:100 in LB supplemented with ampicillin and arabinose and grown for 6 hours at 37°C with shaking. Bacterial cultures were harvested by centrifugation. The resulting cell pellet was resuspended in 20 mM sodium phosphate buffer containing 500 mM NaCl and 20 mM imidazole and lysed via French pressure cell. Bacterial lysates were centrifuged at 20,000 × *g* to remove cellular debris. Clarified supernatants were fractionated using a BioRad BioLogic FPLC system equipped with a HisTrap FF nickel affinity column (GE Healthcare, Piscataway, NJ) over a 20–500 mM linear imidazole gradient. It was determined that the TadD protein eluted between 100 and 200 mM imidazole.

### NAD(P)H-dependent glyceraldehyde 3 phosphate activity assay

NP-G3PDH activity was assayed as previously described with minor modifications [45]. Briefly, reactions contained 1 mM NAD(P)^+^, 1 mM glyceraldehyde 3-phosphate and either 10 μg of purified TadD or 100 μg of crude bacterial lysate in a final volume of 1 ml. Reactions were incubated at 37°C for one hour. Following incubation the optical density of the reactions was measured at 340 nm. *R. opacus* cultures were grown in either lysogeny broth or MR medium supplemented with 4% (w/v) glucose and either 1% (w/v) or 0.15% (w/v) ammonium sulfate for 24 hours prior to mechanical disruption using a French pressure cell. Cellular debris was pelted by centrifugation at 20,000 × *g* for 30 minutes.

### Determination of NAD^+^/NADH and NAD(P)^+^/NAD(P)H ratios

NAD^+^, NADH, NAD(P)^+^ and NAD(P)H concentrations were determined using the Biovision NAD^+^/NADH quantification and the Biovision NAD(P)^+^/NAD(P)H quantification kits, respectively (Mountain View, CA) per manufacturer’s instructions. Cell lysates of *R. opacus* PD630 were prepared as described above.

## Competing interests

The authors declare that they have no competing interest.

## Authors' contributions

DM participated in the conceptualization and design of the studies, performed all experimental work and drafted the manuscript. AS participated in conceptualizing the studies, coordinated undertakings and aided in editing the manuscript. All authors read and approved the final manuscript.
